# Correction for Van Fossen et al., “Profiling sorghum-microbe interactions with a specialized photoaffinity probe identifies key sorgoleone binders in *Acinetobacter pittii*”

**DOI:** 10.1128/aem.00810-25

**Published:** 2025-06-05

**Authors:** Elise M. Van Fossen, Jared O. Kroll, Lindsey N. Anderson, Andrew D. McNaughton, Daisy Herrera, Yasuhiro Oda, Andrew J. Wilson, William C. Nelson, Neeraj Kumar, Andrew R. Frank, Joshua R. Elmore, Pubudu Handakumbura, Vivian S. Lin, Robert G. Egbert

## AUTHOR CORRECTION

Volume 90, no. 10, e01026-24, 2024, https://doi.org/10.1128/aem.01026-24. Supplemental material: The file containing supplemental methods and figures was missing. The file is included in this correction.

## SUPPLEMENTAL MATERIAL

Supplemental material aem.00810-25-s0001.docx Supplemental methods and Fig. S1 to S8.

ASM does not own the copyrights to Supplemental Material that may be linked to, or accessed through, an article. The authors have granted ASM a non-exclusive, world-wide license to publish the Supplemental Material files. Please contact the corresponding author directly for reuse.

